# Action Planning Mediates Guidance of Visual Attention from Working Memory

**DOI:** 10.1155/2015/387378

**Published:** 2015-06-18

**Authors:** Tobias Feldmann-Wüstefeld, Anna Schubö

**Affiliations:** Philipps-University Marburg, 35037 Marburg, Germany

## Abstract

Visual search is impaired when a salient task-irrelevant stimulus is presented together with the target. Recent research has shown that this attentional capture effect is enhanced when the salient stimulus matches working memory (WM) content, arguing in favor of attention guidance from WM. Visual attention was also shown to be closely coupled with action planning. Preparing a movement renders action-relevant perceptual dimensions more salient and thus increases search efficiency for stimuli sharing that dimension. The present study aimed at revealing common underlying mechanisms for selective attention, WM, and action planning. Participants both prepared a specific movement (grasping or pointing) and memorized a color hue. Before the movement was executed towards an object of the memorized color, a visual search task (additional singleton) was performed. Results showed that distraction from target was more pronounced when the additional singleton had a memorized color. This WM-guided attention deployment was more pronounced when participants prepared a grasping movement. We argue that preparing a grasping movement mediates attention guidance from WM content by enhancing representations of memory content that matches the distractor shape (i.e., circles), thus encouraging attentional capture by circle distractors of the memorized color. We conclude that templates for visual search, action planning, and WM compete for resources and thus cause interferences.

## 1. Introduction

Due to the limited capacity of our visual system, selective attention needs to efficiently filter relevant stimuli from the incoming stream of visual information. Which stimuli are selected for further processing is a complex process that depends on various factors such as physical salience (e.g., [1, 2]), task-relevance [3, 4], threat relevance [5, 6], or learning experience [7]. Current visual attention theories, modeling which information is selected under which circumstances, often assume that the visual system weights such factors and assigns priority to each stimulus in the visual field, determining the probability with which it is selected [8–11].

Naturally, as observers, we desire to attend to those stimuli that correspond to our current intentions and goals. By means of a* search template* that directly refers to a mental image of the desired object we can efficiently find such stimuli and deploy our attentional resources to it [12–14]. High weights for features corresponding to current goals of the observer can also have detrimental effects, impairing performance. For example, “contingent capture” describes the effect that visual attention is captured by task-irrelevant stimuli that match the search template in one or more features [15, 16]. It was argued that attention deployment towards stimuli that partially match the search template, regardless of its actual task-relevance, is mediated by Working Memory (WM) which may store the search template so that the observer can constantly match incoming information in ongoing tasks [8, 17–20]. A study by Woodman & Arita [21] provided neurophysiological evidence that attentional templates are maintained in WM and that the strength of template representations in WM predicts the performance in a visual search task.

As WM has a limited capacity, search templates maintained in WM for a search task at hand may sometimes compete with other information maintained in WM. More recent research has indeed suggested that visual selection can be closely intertwined with visual working memory processes [22–25], for example, by showing memory-driven attentional capture. For example, Olivers and Eimer [23] presented observers colored items they had to maintain in WM before they were being probed for correct retrieval. During maintenance, observers performed a visual search task. When distractors shared the colors of items maintained in WM, search was slowed down compared to distractors of neutral colors, suggesting attentional capture by features in WM (see also [24, 26]).

Although it seems plausible that our perception can be biased towards stimuli that match our goals and intentions, perception as such is never a self-purpose; we intend to perceive something so that we can* act* upon the perceived. Action as the purpose of selection has been brought forward by Allport's* selection-for-action* theory Allport [27] and a large body of literature has pointed towards a close interrelation of selection and action since (e.g., [28–34]). For example, in Wykowska et al.'s [32] study, participants had to prepare a grasping or pointing movement according to a cue presented in the beginning of each trial. For grasping, size is a relevant dimension because grasping requires the specification of size-related parameters to control grip aperture. For pointing, luminance is a relevant dimension because pointing crucially requires the localization of to-be-pointed objects—and this localization is efficiently enabled by luminance [33, 35].

Before participants executed the movement, they were shown a search display with various filled grey circles. Either all circles were identical, or one circle was a size singleton (smaller circle) or one circle was a luminance singleton (brighter circle). Participants had to report whether a singleton was present or not. After the search task, participants executed the previously prepared movement towards one among various, real objects in front of them. It was found that preparing a grasping movement facilitated detection of size singletons, whereas preparing a pointing movement facilitated detection of luminance singletons. The authors concluded that planning an action biases visual perception towards dimensions that deliver important information for controlling that action [32]. The large overlaps between visual attention and WM have been attributed to attentional control mechanisms being in some way common with those recruited in the service of WM maintenance [23, 25]. Similarly, the overlaps between visual attention and action planning have been ascribed to a common coding of perception and action [29, 32, 36]. Are WM and action planning also interrelated? It seems economically plausible that a common mechanism allows the visual system (a) to deploy attention to the relevant information currently available to the senses, (b) to maintain information not present to the senses anymore, and (c) to plan action related to the present/maintained visual information. This study aimed at investigating such interactions of visual attention, visual WM, and action planning. More specifically we were interested in whether attention is particularly biased towards dimensions that are both action relevant and WM relevant, for example, towards a red circle if red is to be held in WM and a grasping movement is to be executed towards a circular item.

*Rationale of the Present Study*. The present study combined a visual search task with a working memory (WM) task and a motor task. Participants had to prepare a pointing or grasping movement but withhold execution until the end of a trial. Participants also had to memorize a color hue until the end of a trial. At the end of a trial, participants performed the planned movement towards the memorized (circular) item, thus combining WM and motor planning. While participants planned the movement and held the color in their WM, they had to perform a visual search task in which they always had to respond to a diamond-shaped target among circle distractors. Note that, for grasping, shape was a relevant dimension because the to-be-grasped objects were circles and thus required the specification of circle-related parameters (e.g., [37, 38]). In some of the trials, one of the distractors could be colored; the color could be related or unrelated to the color in WM. We hypothesized that the additional color singletons will capture attention and slow down response times (c.f., [2]), even more so when they are related to WM content (c.f., [23]). Memory-relevance and action-relevance coincided when the additional singleton in the search task was of a color related to WM and when a grasping movement was being prepared. Thus we expected the capture effect to be most pronounced when the color singleton both matches the WM content and is congruent to the action-relevant dimension, because, for such stimuli, task-relevance and action-relevance coincided.

## 2. Method

### 2.1. Participants

21 volunteers (aged 19–27 years) naive to paradigm and objective of the experiment participated for course credit. All but two were right-handed and all had normal or corrected-to-normal vision (tested with Landolt ring test, visus ≥ 1). The experiment was conducted with the understanding and consent of each participant.

### 2.2. Apparatus and Stimuli

Participants were seated in a comfortable chair in a dimly lit room at 50 cm distance from an LCD-TN screen (*Samsung Syncmaster 2233*). Participants had a customizable keyboard (*Ergodex DX1*) on their left side in front of them with two buttons that were labeled “*M*” and “*N*” and were operated by the middle and index finger of the left hand. Stimulus presentation and response collection were controlled by a* Windows* PC using* E-Prime 2* routines. The examiner sat approximately 2 m behind the participant with a standard keyboard on her lap to register participants' movement.

**Figure 1:**
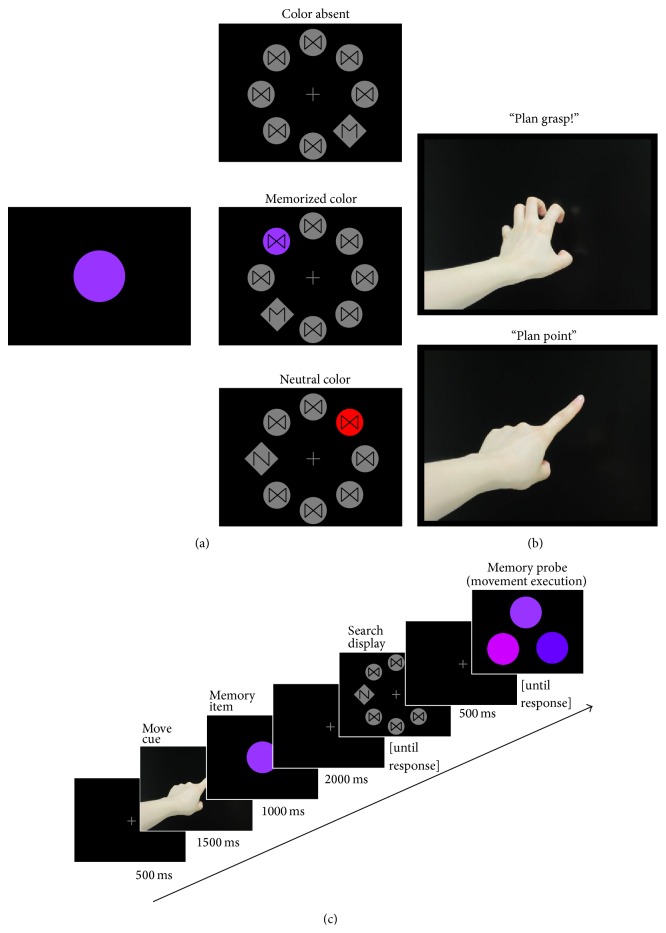
(a) shows the three color conditions used in the present study. The memory item was a centrally presented filled circle (left side) that had one out of thirty-six color hues. Participants had to remember the hue until the end of a trial. In the search task (right side), participants had to report the letter (“*M*” or “*N*”) embedded in the grey diamond-shaped target. Distractors had an embedded tilted hourglass. In the “color absent” condition all distractors were grey. In the “memorized color” condition, one of the distractors had a hue from the same color category as the memory item previously shown. In the “neutral color” condition, one of the distractors had a hue from another color category. (b) shows the movement cues. When a grasping hand was shown, participants had to plan a grasping movement (upper picture). When a pointing hand was shown, participants had to plan a pointing movement (lower picture). Execution of the movement had to be withheld until the end of the trial. (c) shows a trial procedure. Participants had to plan a movement (move cue) and remember a color hue (memory item) while performing a visual search task (search display). After search was completed, participants had to perform the planned movement towards the colored item they thought would match the original memory item.

Movement cues were color photos taken from a female volunteer showing a forearm/hand on a black background that performs a pointing or grasping movement (see Figure 1(b)). The numbers of pixels for the pointing and grasping hands were approximately identical (~20% of the screen; ~80% were black pixels). Memory items were filled circles (diameter: 3.6° visual angle) colored in one of nine shades of four colors (blue, green, yellow, and red), that is, 36 different hues in total. RGB values were identical to those used by Olivers & Eimer [23]; see the appendix for RGB and CIE(*x*, *y*) values. Search displays consisted of eight items placed on an imaginary circle with a diameter of 10.6° (see Figure 1(a)). Distractors were filled circles (2.4°) and could be grey or colored. Colors were taken from the same pool of colors as memory items (see above). Grey and colored distractors had an embedded tilted black hourglass. Targets were grey diamonds (3°) with an embedded stylized black *M* or *N*. *M* and *N* were made by removing two lines from the hourglass; that is, *M*, *N*, and hourglass were superposable. In* color absent* trials, there was one target and seven grey distractors. The target was presented equally often in each of the eight positions. In* memorized color *trials and* neutral color* trials, there was one target, one color distractor, and six grey distractors. Target and color singleton were presented equally often in each of the eight positions but never in neighboring positions.

### 2.3. Procedure

Each trial started with a central fixation, shown for 500 ms (see Figure 1(c)), and was replaced with a movement cue shown for 1500 ms. Participants were instructed to prepare the indicated movement but withhold movement execution until the end of the trial. A display followed for 1000 ms that randomly showed one of the 36 color hues. Participants were instructed to memorize this hue until it was probed in the end of the trial. After a blank display showing only a fixation cross, the search display was shown. In one-third of the trials (320 trials), all distractors were grey (color absent). In another third of the trials, a color distractor was presented that had a hue from the same color category as the memorized item but never the exact same hue (memorized color). In the remaining third of the trials, a color distractor was presented that had a hue from a different color category as the memorized item (neutral color). Participants were instructed to respond as fast as possible (while avoiding errors) to the letter embedded in the target (50% *M*, 50% *N*) by pressing the according button on the keyboard. After participants responded, the search display was replaced by another blank display for 500 ms. Subsequently, a memory probe display appeared with three (circular) memory items arranged in a pyramid arrangement. Participants were instructed to execute the planned movement towards the item with the memorized hue. The two other items were always from the same color category as the memorized hue (e.g., if the memorized items were from the category “red,” two out of the remaining eight hues were randomly selected to appear together with the memorized hue). All items being from the same color category was thought to discourage verbalization of the color and encourage a visual memory representation. For pointing movements participants had to touch the screen with the tip of their right index finger. For the grasping movement participants had to make a claw-like gesture with their right hand, touching the screen with all five fingers along the outlines of the respective memory item. After participants performed the movement, the examiner pressed one out of two buttons to register which movement had been executed by the participant (point versus grasp) and pressed one out of three buttons to register towards which item the movement was made (first, second, and third colored circle within the pyramid arrangement). Only trials in which participants performed the correct movement and towards the correct object were counted as correct trials. After an intertrial interval of 1000 ms (black screen), a new fixation cross announced the beginning of a new trial.

There were six experimental conditions: three color conditions (color absent, memorized color, and neutral color) and two movement conditions (pointing and grasping). Experimental condition was randomly chosen in each trial. Target position (or target-color distractor position combination, resp.) was counterbalanced with each of the six experimental conditions across the experiment. The experiment comprised 960 trials (+30 practice trials) divided in 40 blocks of 48 trials, that is, 160 trials per experimental condition. After each block a pause of at least 10 sec followed and performance feedback (RTs and accuracy for the search task and accuracy for the motor task) was provided to participants on the screen.

### 2.4. Data Analysis

Mean response times (RT) and error rates were calculated for each participant separately for each of the six experimental conditions. For “memorized color” trials, RTs were collapsed across all hues from the same category as the memory item. For “neutral colors,” RTs were collapsed across all hues from all categories to which the memory item did not belong. Only trials in which all three tasks (search task, motor task, and memory task) were performed correctly and trials without exceedingly long search RTs (>2000 ms) or short RTs (<300 ms) remained in the RT analyses. These criteria were reached in 64.5 % (SD = 8.8%) of the trials. Data were submitted to a 2 × 3 ANOVA with the factors color (absent versus memorized versus neutral) and movement (pointing versus grasping). Greenhouse-Geisser correction was applied when appropriate. As measures of effect size, partial eta squared (*η* ^2^) is reported for ANOVAs, and epsilon (*ε*) is reported for *t*-tests.

## 3. Results

### 3.1. Search Task

**Figure 2:**
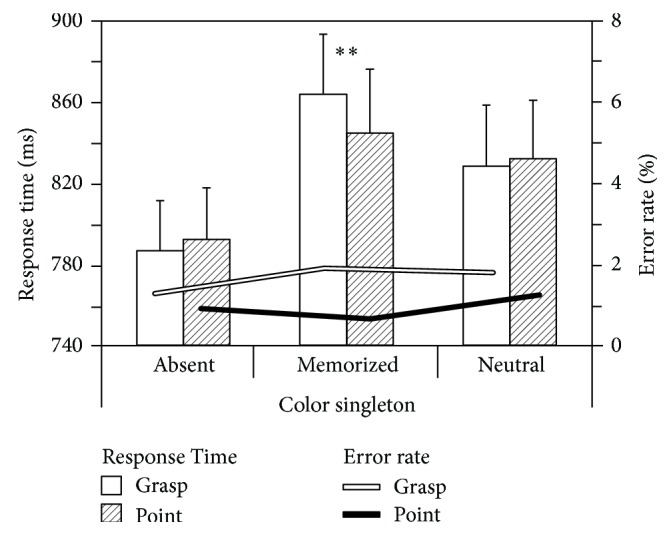
Response times (left *y*-axis) and error rates (right *y*-axis). White bars and lines represent performance in trials in which participants planned a grasping movement. Shaded bars and black lines represent performance in trials in which participants planned a pointing movement. Asterisks (*∗∗*) indicate a *p* value ≤ 0.01 for a direct comparison of grasp and point. Error bars represent standard errors of the mean.

Search RTs depended on the presence of a color distractor, *F*(2,40) = 32.96, *p* < 0.001, *η* ^2^ = 0.622. RTs were shortest when color was absent (*M* = 789 ms) followed by distractors with neutral colors (*M* = 830 ms) and distractors with memorized colors (*M* = 854 ms), all *p* < 0.001 (contrasts); see Figure 2. How much search RTs were modulated by color singleton depended on preparation of a movement, *F*(2,40) = 5.44, *p* = 0.008, *η* ^2^ = 0.214. Follow-up *t*-test for dependent measures showed that differential movement preparation (pointing versus grasping) did not affect neither trials without color distractors (Δ*M* = 6 ms), *t*(21) = 0.79, *p* = 0.440, *ε* = 0.24 nor trials with neutral color distractors (Δ*M* = 4 ms), *t*(21) = 0.61, *p* = 0.548, *ε* = 0.19. In trials with memorized color distractors, however, preparing a grasping movement increased search RTs compared to preparing a pointing movement (Δ*M* = 19 ms), *t*(21) = 2.85, *p* = 0.010, *ε* = 0.88. To confirm that the 2-way interaction is not a merely additive effect of WM and action planning, an additional ANOVA was calculated in which the capture effect was taken as a dependent measure (mean RTs for “color absent” were subtracted from mean RTs for “memorized color” and “neutral color”, resp.). A 2 × 2 ANOVA with the factors color (memorized versus neutral) and movement (pointing versus grasping) was then run. Results showed that the capture effect was not modulated by the preparation of a movement (*p* = 0.084) but was modulated by the distractor type (*M*_mem_ = 65 ms versus *M*_neutr_ = 41 ms), *F*(1,40) = 22.59, *p* < 0.001, *η* ^2^ = 0.530. The increase in attentional capture for memorized colors was more pronounced for grasping (Δ*M* = 35 ms) than for pointing (Δ*M* = 13 ms), *F*(1,40) = 7.72, *p* = 0.012, *η* ^2^ = 0.279. ANOVA for error rates revealed no effects (all *p* ≥ 0.437).

### 3.2. Motor Task

The correct movement according to the movement cue was executed equally often for grasping (93.3%) as for pointing (92.3%), *p* > 0.5.

### 3.3. Memory Task

**Figure 3:**
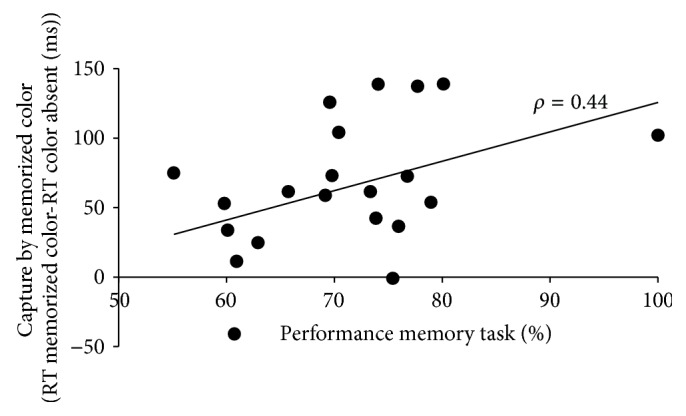
Scatter plot showing the bivariate correlation between the capture by memorized colors and the performance in the memory task. Capture is defined by the RT increase in trials with distractors with memorized colors compared to trials with no colored distractor. Each black circle represents one participant. The pattern of results shows that participants that were good in memorizing colors in the working memory task were more likely distracted by memorized colors in the search task.

The memory items were correctly identified (in terms of executing the movement towards the item of the hue that was shown in the beginning of the trial) in 71.0% of the trials (SD = 2.1%). To reveal whether those participants that were best in memorizing were also the ones that were most distracted by memorized color distractors, Pearson's product-moment correlation was computed for memory accuracy and RT difference for color absent versus* memorized* color (across movement conditions). Results showed a strong correlation (*ρ* = 0.44; *p* = 0.024); that is, the better memory performance, the more RT impairment due to memorized colors; see Figure 3. No such correlation was found for memory performance and RT impairment due to* neutral* colors (*p* = 0.127).

## 4. Discussion

The key result of the present study is an interrelation of action planning and maintenance in working memory (WM). Attention was particularly biased towards colors matching WM content when participants prepared a grasping movement compared to a pointing movement. When no color distractor was presented or the color distractor had a color unrelated to WM content, the preparation of the movement did not affect attention deployment.

The presence of a color distractor impaired visual search even though colors were entirely search-irrelevant. Even though the task could be solved and the target be found most of the times (overall error rates ~3%), response times were slowed down. This is a replication of earlier findings showing that salient stimuli can capture attention against the intention of an observer [2, 39–41]. Apparently, salient distracting information needs to be rejected before attention can be directed towards the task-relevant stimulus, a time demanding process. Current visual attention models account for attentional capture, for example, by assuming that the weight of any bottom-up feature map cannot be set to zero by the observer, regardless of the task-relevance of the feature dimension (*Guided Search*, [11, 14]). By this means, salient stimuli will always project on the master map of activations (or,* priority map*, [9]), based on which attention deployment is being made.

The present study also showed that the attentional capture was particularly pronounced when the color distractor matched the color category that was held in WM. This suggests that stimuli matching information maintained in WM are given more priority in visual processing than stimuli of equal salience that were not maintained in WM.

It should be noted that the longer response times for trials in which task-irrelevant color distractors were present do not necessarily indicate that those distractors were processed in a bottom-up manner. According to Bacon and Egeth [51], two search modes would have been applicable in the present search task:* singleton detection* mode in which observers search for anything that stands out from the background and* feature search* mode in which observers search for a predefined feature (here, diamond), a search mode only possible when the target feature is known and does not change between trials. As in the present paradigm observers searched for a diamond-shape target throughout the experiment while ignoring color both search modes could have been used. Applying the feature search mode (rather than singleton detection) would have been particularly beneficial to color-present trials as distraction by the irrelevant color singleton would have been minimized. In color absent trials, however, observers could have applied the simpler singleton detection mode as this search mode was sufficient to efficiently find the target. If this was the case longer RTs for color-present trials may have been due to differences in search strategy and not to bottom-up attentional capture. Different search modes in color-absent and color-present trials seem, however, rather unlikely as the trial types were completely randomized between trials. This makes it unlikely that observers switched their search mode between trials because they could not predict which trial type would follow. Only as soon as stimuli showed up on the screen could observers know whether a color was present or not.

Regardless of the applied search mode, more priority to WM-related stimuli is well in line with the notion that visual attention and WM may rely on the same mechanism of prioritization of items [52–54] and replicates earlier results showing that guidance of attention from WM occurs even when it is detrimental to performance [13, 23, 25, 55]. For example, Olivers and colleagues [13, 23] argue that search templates and other representations in WM compete for resources. As a search template, items have full access to the sensory input and can bias selection towards matching objects. Maintaining an item in WM (e.g., because it will be probed in the end of a trial) is similar to adopting a search template (or an “attentional set”; [23]). Such representations (so-called “accessory memory items”) have a passive status after being encoded into WM to not interfere with an ongoing task (here, the search task) and can be reactivated to regain the status of a search template when they become relevant (here, when memory probes are presented). Reactivation of accessory memory items may also happen involuntarily, for example, when a WM matching distractor is presented during the search task. This leads to prioritization of features held in WM, attentional capture results which may impair performance (see also [24, 25]). In the present study this was the case in the “memorized color” condition. In terms of salience maps, it seems that weights for features in the WM task spill over to visual search and bias attention accordingly (e.g., [53, 56]).

The increased attentional capture by color distractors matching the item held in WM could also be due to color priming from the WM displays to the search displays [7, 57]. Previous studies using a similar paradigm, however, could rule out the possibility of priming effects (e.g., [24, 26, 58]). Those studies showed that attentional capture during a search task occurred only when previously presented items had to be remembered. When observers were merely exposed to the crucial features, no such effects were found. Additional evidence comes from an imaging study [59] when WM content was repeated in a search task, enhanced activity in a variety of brain regions known to be sensitive to the prior history of events was found. Conversely, when items only had to be identified but not held in WM, their repetition elicited a suppressed response in the same regions. Given the similar paradigm it seems likely that, also in the present experiment, visual search costs were due to WM content rather than repetition effects.

**Figure 4:**
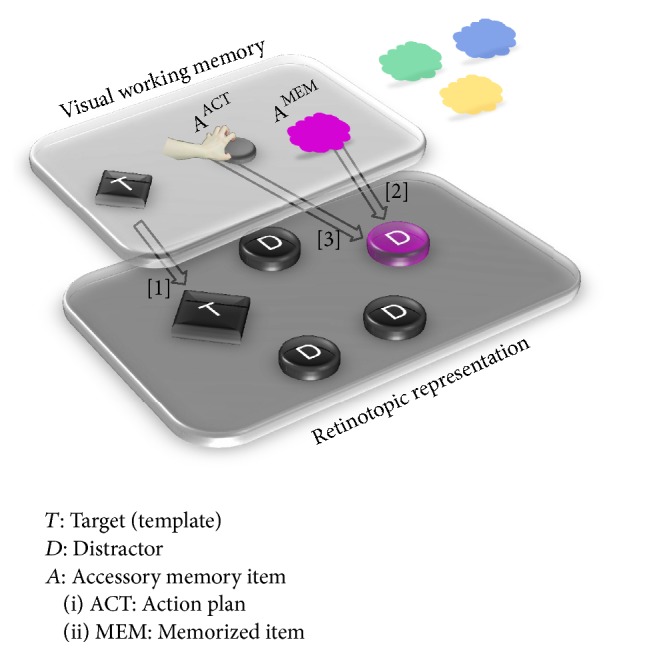
Potential extension of a visual working memory (WM) model suggested by Olivers et al. [13] to account for the interactions between working memory and action planning in the present study. A representation of the target (*T*) is kept in WM in form of a search template that facilitates finding targets in visual search displays [1]. Accessory memory items (*A*) are representations of objects that are in principle accessible to the visual system but are currently not relevant for the task. In the current study, accessory memory items are color hues to be remembered at the end of a trial but completely irrelevant in the search task (*A* ^MEM^). In “memorized color” trials they can impair search performance by enhancing an irrelevant distractor (*D*) that shares their color category [2]. Our data suggest that distraction from target by objects of memorized colors is more pronounced when a grasping (rather than a pointing) movement is being prepared. We hypothesize that maintenance of information for subsequent action execution (“action plan”) induces an accessory memory item for action relevant features (*A* ^ACT^). As the to-be-grasped object is a circle, circular distractors with memorized colors receive further enhancement, thus increasing further interference with the target [3].

Interestingly, the present results indicate that guidance of attention from WM was most pronounced when participants prepared a grasping movement. This indicates that apart from overlaps between visual attention and WM there may also be a connection between WM and action planning; see WM model in Figure 4. There is quite some evidence that action planning has a strong impact on perceptual processes, even in situations when action and perception are not directed to the same objects but merely overlap in time (e.g., [28–34]). For example, when observers have the intention to perform an action, such as grasping or pointing, their attention can be biased towards simultaneously presented yet unrelated visual objects. This leads to faster target detection when the target is congruent to the planned action compared to incongruent targets [32, 34].

In the present study, the feature dimension “shape” was crucial to the search task; the feature “diamond” was task-relevant as targets were diamond-shaped; the feature “circle” was task-irrelevant as distractors were circle-shaped. Shape is a dimension also relevant to grasping as grasping requires the specification of shape-related parameters. For example, for a safe grip of an object, an agent needs to know the properties of its surface; a ball is grasped differently than a cube (e.g., [37, 38]). In the present study, participants had to grasp circular items in the memory probe display, rendering the feature “circle” particularly crucial for action planning. Conversely, when participants had to point at the circular items, no feature should have been rendered more crucial because none of the features in the search task (shape, color) were particularly relevant for pointing. Our results show that movement planning modulates attention guidance from WM. It appears that when circles were made relevant through planning of a grasping movement, circular distractors captured more attention than when a pointing movement was planned but only if their color matched WM content. Thus WM might be a mediating process involved in the relation of action planning and attention. In terms of the WM model by Olivers and colleagues the planning of an action such as a grasping movement might increase the demand on WM and thus cause additional interference in WM because it requires the maintenance of information for subsequent action execution (see Figure 3). In the present study preparing a grasping movement may have needed the maintenance of an action-relevant feature “circle shape” in WM. This action-relevant feature may have prioritized processing of circle distractors in memorized colors in the search task, thus increasing interference with target processing and impairing performance.

Our data show that action planning only modulated search performance when participants prepared a grasping movement* and* one of the distractors was in a memorized color. This suggests that only when both* circle prioritization* by action-planning and* color prioritization* by WM maintenance coincided did attentional capture by the color singleton particularly impair search performance. This is strong evidence for a common mechanism underlying selective attention, WM, and action planning. The present results thus add to the previous literature showing that action planning is a rather dominant source of top-down control in selection and not only can modulate attention deployment but also can interact with WM content.

Interestingly, participants differentiated hues in the memory task quite well. Even though nine hues had to be distinguished within one color category, participants correctly remembered the hue in ~71% of the cases (chance level 1/3). If the ability to discriminate hues was as good for color distractors as for memory probes, one would expect no distraction away from the target as the color singleton never exactly matched the hue of the memorized color. However, hues within the same color category induced a significant increase in target search time compared to a neutral color category. This indicates that, whereas participants were able to keep a relatively precise representation of the memory item in WM, a much broader variety of hues, actually the entire color category, was able to involuntarily capture attention. Perception of similar hues as one color category is a basic mechanism of the visual system [60, 61] and is, for example, crucial to perceive a constant surface under varying lighting conditions [62]. In the present study, whether hues were perceived as belonging to one category or not might have been due to varying time pressure or processing strategies. For example, observers may have been biased towards the entire WM-relevant color category under the time pressure of the search task (RT was stressed for the search task) that did not allow the visual system to differentiate between hues. When the memory probe was shown in the end of a trial, however, observers were able to make more precise color judgments (precision was stressed for the memory task). Moreover, the visual system may have relied on color categories when no explicit incentive to differentiate between hues was present (such as for task-irrelevant distractors during search); thus attention was biased towards the entire category. This may in fact be an adaptive strategy of the visual system to cope with the invariance of physical features in real world settings. For example, “color constancy” describes the effect that observers perceive the color of an object constant even though the exact wavelength may change drastically as a function of environmental variables such as illumination, perspective, shading, and distance [63, 64]. More recently it was found that attention deployment can be biased towards perceptual categories that typically share very few low-level features, even when this is detrimental to performance [65]. The present results may provide an example in which attention deployment is biased towards a color category even though only one subset of that category (a specific hue) was task-relevant. A strategy to more thoroughly process colors, however, might have been used when observers had the incentive to correctly identify one hue among similar ones (such as for the memory probe). This notion is in line with findings suggesting that how prominent the effect of color category on hue perception is relies on various factors such as the specific task, cognitive demands, and strategies of the observer [66].

In the present study we argue that the additional singleton captured attention against the will of the observers. It should be noted that some studies suggest that longer response times for distractor-present trials in additional singleton paradigms are due to nonspatial filtering costs [42–45] whereas other studies suggest they are due to attentional capture by the distractor [39, 46–48]. One reason for the diverging results may be that singletons only capture attention when they match the observer's goal [49]. Although the present study cannot contribute to answering this open question, we assume that in the present study attentional capture accounts for the longer response times for distractor-present trials. Even though color distractors never matched the target category, they did match the WM content, namely, in the “memorized color” condition. As it was the observers' goal to maintain items in WM, attentional capture by items matching WM content may therefore be considered attentional capture due to matching of the observer's goal [49]. In line with this, in a study using a similar paradigm as the present study, an N2pc component contralateral to the memory-matching distractor (i.e., a distractor negativity) was found, suggesting that indeed stimuli sharing features of WM content capture attention [50].

## 5. Conclusions

To sum up, the present results showed that action planning not only determines the way we perceive our visual environment but also determines how working memory (WM) drives attention deployment. Observers were distracted from target search when a salient color item was presented concurrently to the target. This distraction was more pronounced when the colored item was related to a color held in WM, indicating attention guidance by WM content. Preparing a grasping movement enhanced WM guided attention deployment. We conclude that target templates, action plans, and WM maintenance have common underlying mechanisms and compete for processing capacity.

## Figures and Tables

**Table 1:** 

	Red 1	Red 2	Red 3	Red 4	Red 5	Red 6	Red 7	Red 8	Red 9
R	239	228	216	239	228	217	237	227	216
G	30	55	70	33	56	71	43	60	73
B	82	86	89	63	69	75	42	51	60
CIE(*x*)	0.574	0.546	0.522	0.596	0.569	0.540	0.609	0.583	0.558
CIE(*y*)	0.312	0.319	0.326	0.327	0.334	0.341	0.343	0.350	0.355

	Blue 1	Blue 2	Blue 3	Blue 4	Blue 5	Blue 6	Blue 7	Blue 8	Blue 9

R	0	42	65	90	95	100	107	109	111
G	121	121	121	111	113	115	108	111	113
B	234	218	203	230	216	202	227	214	200
CIE(*x*)	0.175	0.183	0.193	0.201	0.211	0.223	0.215	0.224	0.234
CIE(*y*)	0.173	0.194	0.198	0.176	0.194	0.213	0.180	0.196	0.215

	Green 1	Green 2	Green 3	Green 4	Green 5	Green 6	Green 7	Green 8	Green 9

R	91	97	104	71	82	93	46	68	84
G	134	132	130	137	134	131	139	136	133
B	0	39	63	29	54	73	50	67	81
CIE(*x*)	0.335	0.340	0.337	0.305	0.311	0.314	0.272	0.285	0.295
CIE(*y*)	0.551	0.524	0.482	0.560	0.518	0.473	0.552	0.505	0.464

	Yellow 1	Yellow 2	Yellow 3	Yellow 4	Yellow 5	Yellow 6	Yellow 7	Yellow 8	Yellow 9

R	254	248	242	245	240	234	236	229	225
G	190	192	193	194	195	197	194	199	200
B	37	74	101	8	66	96	0	57	90
CIE(*x*)	0.458	0.439	0.419	0.449	0.433	0.412	0.440	0.422	0.405
CIE(*y*)	0.466	0.455	0.439	0.478	0.465	0.450	0.484	0.479	0.461

Color definitions for the 36 hues used for the search and memory task. RGB (red, green, and blue) values and CIE(*x*, *y*) values, measured with the *HCFR* Monitor Calibration Software (http://sourceforge.net/projects/hcfr/) using an *x-rite i1 DisplayPro* colorimeter, are specified.

## Appendix

See (Table 1).
